# The lactate-to-albumin ratio as a potential biomarker for short-term mortality risk in critically ill patients with urosepsis: a retrospective study with dual-cohort validation

**DOI:** 10.3389/fnut.2026.1753403

**Published:** 2026-02-17

**Authors:** Xiangqian Nie, Zhenlin He, Kun Wang, Decai Zhu, Lei Zhang, JianXin Hu, Ying Yu

**Affiliations:** 1 Department of Urology, Zhejiang Provincial People’s Hospital Bijie Hospital, Bijie, China; 2 Department of Urology, Guizhou Provincial People’s Hospital, Guiyang, China

**Keywords:** adverse prognosis, biomarker, lactate-to-albumin ratio (LAR), risk stratification, urosepsis

## Abstract

**Background:**

The lactate-to-albumin ratio (LAR) comprehensively reflects the nutritional status, inflammatory level, and degree of oxidative stress in critically ill patients and has been demonstrated to be associated with poor prognosis in various critical illnesses. However, its prognostic role in the specific context of urosepsis remains to be fully elucidated.

**Methods:**

This study utilized the Medical Information Mart for Intensive Care (MIMIC-IV) database as an internal discovery cohort and included an external validation cohort from Bijie Hospital of Zhejiang Provincial People’s Hospital. Multivariable Cox regression, restricted cubic splines (RCS), Kaplan–Meier survival curves, and other methods were employed to analyze the association between LAR and short-term adverse outcomes in urosepsis patients. The robustness of the findings was assessed through subgroup analysis and interaction tests. Furthermore, four machine learning algorithms were combined to screen key variables for constructing a multivariable Cox risk prediction model, the performance of which was evaluated using receiver operating characteristic (ROC) curves.

**Results:**

A total of 1,055 urosepsis patients were included. The 28-day ICU mortality and in-hospital mortality rates were 20.6 and 19.1%, respectively. After multivariable adjustment, for each unit increase in LAR as a continuous variable, the hazard ratios (HR) for ICU mortality and in-hospital mortality were 1.35 (95% CI: 1.08–1.68) and 1.50 (95% CI: 1.19–1.90), respectively. When treated as a categorical variable, the high LAR group had a 73% increased risk of ICU mortality and a 71% increased risk of in-hospital mortality compared to the low LAR group. RCS analysis revealed a non-linear positive dose–response relationship between LAR and mortality. ROC analysis demonstrated that a simple risk model incorporating LAR and five other variables had superior predictive performance compared to traditional critical illness scores, and these conclusions were consistently validated in the external cohort.

**Conclusion:**

The LAR is a cost-effective supplementary predictor of short-term mortality risk in critically ill urosepsis patients, primarily aiding early identification of high-risk cases to intensify monitoring and treatment. Its clinical value require further prospective validation.

## Background

Sepsis, a life-threatening systemic inflammatory response triggered by infection, has become the leading cause of mortality in non-cardiac intensive care units (1, 2). Its pathological mechanisms demonstrate source-specific characteristics: pulmonary infections primarily feature alveolar barrier disruption and macrophage overactivation, often progressing to acute respiratory distress syndrome; abdominal infections are characterized by bacterial translocation and frequently lead to intra-abdominal hypertension; while urosepsis is primarily triggered by Gram-negative bacterial endotoxins that provoke systemic inflammation (3–5). Accounting for 9–31% of all sepsis cases (6, 7), urosepsis presents an increasingly serious clinical challenge due to rising urinary tract disease incidence and growing antibiotic resistance (3, 4). Consequently, identifying risk factors for urinary tract infection-related bloodstream infections and establishing early biomarkers holds crucial clinical significance for advancing precision medicine and improving patient outcomes.

Serum albumin, the most abundant protein in plasma, is an important biomarker for assessing nutritional status and systemic inflammation (8, 9). It helps maintain fluid balance and osmotic stability between intravascular and extravascular compartments and serves as a carrier for various substances including hormones, drugs, and trace elements (8–10). Studies have shown that low albumin levels are closely associated with functional decline, the occurrence of multiple diseases, and all-cause mortality (11–14). Lactate, a product of anaerobic metabolism, increases significantly under conditions of tissue hypoxia or hypoperfusion and is therefore commonly used as an indicator of disease severity and prognosis in critically ill patients with trauma, infection, or sepsis (15–17). However, the predictive value of using albumin or lactate alone in complex disease states is limited.

The lactate-to-albumin ratio (LAR) is a composite indicator proposed in recent years. It has demonstrated a better predictive ability for adverse outcomes than either parameter alone in various diseases, including heart failure, acute coronary syndrome, and stroke (18–21). Nevertheless, research on the role of LAR in prognostic assessment of patients with urosepsis remains scarce. Therefore, this study aims to systematically validate its independent predictive value in the specific population of urosepsis, a condition characterized by distinct pathophysiological mechanisms.

## Methods

### Data sources

This study utilized data from two retrospective cohorts. The internal training cohort data were extracted from the publicly available Medical Information Mart for Intensive Care (MIMIC-IV) database, maintained by the Massachusetts Institute of Technology. A detailed description of this database can be found in previous literature (22). The external validation cohort included critically ill patients with urosepsis admitted to Bijie Hospital of Zhejiang Provincial People’s Hospital between January 2020 and January 2025. The use of this cohort was approved by the hospital’s Ethics Committee.

### Study population

This study focused on patients admitted to the ICU with urosepsis. Patient diagnosis was based on criteria established in previous literature (23, 24). Sepsis was defined according to the Sepsis-3 consensus criteria, requiring an increase in the Sequential Organ Failure Assessment (SOFA) score by ≥2 points from baseline. The diagnostic criteria for UTI differed between the two cohorts: the internal cohort was identified using International Classification of Diseases (ICD) diagnostic codes from the MIMIC-IV database, while the external cohort was confirmed by typical clinical presentation (pyuria and/or bacteriuria on urinalysis) and a positive urine culture. Only patients with an initial admission diagnosis of UTI who also met the sepsis-3 criteria were enrolled in the urosepsis cohort. All enrolled patients met the following criteria: (1) age ≥18 years; (2) a hospital and ICU stay of at least 24 h; and (3) the availability of complete serum lactate and albumin measurements taken at ICU admission.

### Variable extraction and endpoint definition

Data extraction was performed using PostgreSQL (v13.7.2), Navicat Premium (v16.0), and Structured Query Language (SQL). The extracted variables were categorized into six groups: (1) demographics; (2) comorbidities; (3) vital signs; (4) laboratory parameters; (5) disease severity scores; and (6) administered treatments (see Supplementary Tables S1, S2 for a detailed list). The primary endpoint of the study was 28-day ICU mortality, with 28-day in-hospital all-cause mortality as the secondary endpoint. For variable imputation, we only included variables with less than 30% missing data. Multiple imputation was conducted using the R “mice” package (v3.16.0). It should be noted that the key exposure variables (LAR and its components, serum lactate and albumin) had no missing data in the final analysis cohort due to the inclusion criteria requiring complete measurements. For other covariates meeting the missingness threshold, we generated 5 complete datasets through multiple imputation with 50 iterations. The imputation model incorporated all variables used in the primary analysis: continuous variables requiring imputation, the complete primary exposure variables (LAR and its components), the complete outcome variable (28-day mortality), and all other complete categorical and continuous covariates. Descriptive statistics of the pooled imputed datasets were consistent with those from the original data, indicating no substantial bias was introduced by the imputation process. This approach strengthens the plausibility of the missing at random assumption by leveraging all available information to predict missing values.

### Association between LAR and endpoints

After conducting hypothesis testing, Cox proportional hazards regression model was used to evaluate the association between LAR and clinical endpoints. To control for potential confounding factors, three progressively adjusted models were constructed: Model 1 was unadjusted; Model 2 was adjusted for demographic characteristics (age, sex, ethnicity, and weight) and baseline comorbidities showing intergroup differences; Model 3 was further adjusted for clinically relevant variables demonstrating significant differences between survivors and non-survivors, including severity-of-illness scores, key laboratory parameters, and treatment measures (detailed in Supplementary Tables S1–S3). This comprehensive adjustment approach follows a well-validated strategy consistently employed in previous studies. To mitigate multicollinearity affecting model stability, the variance inflation factor (VIF) was calculated for all variables included in Model 3, and variables with VIF >5 were excluded. Subsequently, restricted cubic splines (RCS) with four knots (at the 5th, 35th, 65th, and 95th percentiles) were used to explore potential nonlinear relationships between LAR and the endpoints. Kaplan–Meier (KM) survival curves were used for supplementary visualization.

### Screening of important prognostic features

The internal cohort was first randomly split into a training set and a validation set in a 7:3 ratio. In the training set, the Boruta algorithm was employed to identify variables associated with short-term ICU mortality. The confidence level (*p*-value threshold) for this process was set at 0.01, with a maximum of 100 iterations for important source runs, and multiple comparison adjustments were performed using the Bonferroni method. Subsequently, three machine learning algorithms were applied to further identify core variables significantly related to patient outcomes from a broader set. The random forest algorithm was configured with 100 trees, a maximum tree depth of 3, a minimum of 2 samples required to split an internal node, and 1 sample required at a leaf node. The GBM was implemented using the Cox proportional hazards loss function (“coxph”). Key parameters included a learning rate of 0.1, 100 boosting stages, a subsample fraction of 1.0, the “friedman_mse” criterion for evaluating split quality, a minimum of 2 samples required to split an internal node, 1 sample required at a leaf node, and a minimum impurity decrease threshold of 0. LASSO regression (least absolute shrinkage and selection operator) was also applied to simultaneously perform feature selection and regularization. Variables identified as important by all three algorithms were considered significant predictors.

### Risk prediction modeling and validation

Following the identification of key variables, these were used to construct a final, interpretable multivariate Cox proportional hazards regression model for risk prediction. The regression coefficients (*β*-values) in the final model formula were directly derived from this Cox model, representing the logarithm of the hazard ratio associated with a one-unit increase in each predictor, after adjusting for all other variables in the model. Each patient’s risk score was calculated using the following formula: Risk Score = (*β*₁ × Variable₁) + (*β*₂ × Variable₂) + (*βₙ* × Variable*ₙ*). The predictive performance of the model was evaluated using receiver operating characteristic (ROC) curves and the area under the curve (AUC). Furthermore, its stability was assessed in an external validation cohort with distinct patient characteristics.

### Statistical analysis

For data visualization and comparative analysis, patients were stratified into low (<0.48718), medium (0.48718–0.84375), and high (>0.84375) LAR groups based on tertiles. It is important to emphasize that this categorization was intended solely for descriptive and exploratory purposes and does not imply the existence of a clinically validated risk threshold. Continuous variables are presented as mean ± standard deviation and were compared using Student’s *t*-test or analysis of variance (ANOVA) after assessing normality and homogeneity of variances. Categorical variables are expressed as numbers (percentages) and were compared using Pearson’s chi-square test or Fisher’s exact test, as appropriate. All statistical analyses were performed using R software (version 4.5.1). A two-sided *p*-value <0.05 was considered statistically significant.

## Results

### Baseline patient data

According to the strict inclusion criteria, 1,055 critically ill patients with urosepsis were included in the analysis. The mean age of the cohort was 70.5 years, and 439 (41.6%) were male. Baseline characteristics stratified by the LAR are presented in Table 1. Compared to patients with lower LAR, those with higher LAR had a higher burden of baseline comorbidities, required more intensive therapeutic interventions, and exhibited less stable vital signs, characterized by an increased respiratory rate and decreased systolic, diastolic, and mean arterial pressures. Furthermore, multiple laboratory parameters differed significantly between the groups. Higher LAR was associated with elevated levels of RDW, WBC, neutrophil count, anion gap, serum glucose, lactate, INR, PT, PTT, ALT, AST, total bilirubin (TB), and lactate dehydrogenase. Conversely, albumin, total serum calcium, total carbon dioxide combining capacity, ionized calcium, pH, and partial pressure of carbon dioxide (PCO₂) were lower in the high LAR group. Clinical severity scores were positively correlated with LAR levels. Mortality was significantly higher in the high LAR group compared to the low LAR group for both 28-day ICU mortality (29.2% vs. 10.4%, *p* < 0.001) and in-hospital mortality (27.2% vs. 9.83%, *p* < 0.001).

**Table 1:** Baseline data of patients in LAR grouping.

	ALL	Low	Moderate	High	*p*-value
	*N* = 1,055	*N* = 346	*N* = 356	*N* = 353	
LAR	0.83 (0.65)	0.36 (0.08)	0.65 (0.10)	1.48 (0.75)	<0.001
Age	70.5 (15.7)	70.0 (15.9)	71.1 (15.1)	70.4 (16.2)	0.663
Gender	439 (41.6%)	149 (43.1%)	151 (42.4%)	139 (39.4%)	0.571
Race	627 (59.4%)	211 (61.0%)	218 (61.2%)	198 (56.1%)	0.292
Weight	82.2 (25.5)	82.5 (25.7)	82.8 (26.3)	81.3 (24.6)	0.721
HTN	354 (33.6%)	114 (32.9%)	119 (33.4%)	121 (34.3%)	0.931
AKI	649 (61.5%)	175 (50.6%)	220 (61.8%)	254 (72.0%)	<0.001
CKD	310 (29.4%)	106 (30.6%)	108 (30.3%)	96 (27.2%)	0.540
DM	401 (38.0%)	126 (36.4%)	131 (36.8%)	144 (40.8%)	0.416
HLD	409 (38.8%)	129 (37.3%)	160 (44.9%)	120 (34.0%)	0.009
HF	659 (62.5%)	211 (61.0%)	218 (61.2%)	230 (65.2%)	0.440
IHD	426 (40.4%)	130 (37.6%)	160 (44.9%)	136 (38.5%)	0.095
COPD	167 (15.8%)	57 (16.5%)	61 (17.1%)	49 (13.9%)	0.456
SOFA	7.06 (3.58)	5.74 (2.98)	7.06 (3.48)	8.37 (3.74)	<0.001
APSIII	56.8 (21.8)	49.6 (18.5)	55.6 (20.1)	65.0 (23.6)	<0.001
SAPSII	44.0 (13.1)	39.6 (12.5)	43.5 (11.8)	48.8 (13.4)	<0.001
OASIS	35.9 (8.42)	33.9 (8.39)	35.8 (8.13)	37.9 (8.30)	<0.001
Charlson	6.03 (2.86)	5.83 (2.92)	6.09 (2.83)	6.15 (2.83)	0.308
APACHEII	21.6 (7.12)	19.5 (6.91)	21.5 (6.73)	23.7 (7.13)	<0.001
HR	90.6 (20.7)	86.0 (20.2)	90.0 (19.2)	95.6 (21.5)	<0.001
NBPS	119 (24.3)	124 (23.2)	119 (24.6)	115 (24.1)	<0.001
NBPD	69.3 (20.2)	71.8 (20.0)	68.5 (19.5)	67.7 (20.9)	0.018
NBPM	82.4 (19.8)	85.4 (19.5)	81.7 (19.3)	80.2 (20.4)	0.002
RR	20.5 (6.66)	20.0 (6.49)	20.7 (6.72)	20.9 (6.73)	0.120
SpO_2_	96.6 (4.56)	96.9 (3.81)	96.4 (4.83)	96.5 (4.95)	0.211
Lym	1.55 (5.58)	1.28 (0.96)	1.35 (1.94)	2.00 (9.39)	0.313
HCT	31.8 (6.94)	32.4 (6.44)	31.4 (6.60)	31.6 (7.69)	0.103
Hb	10.2 (2.28)	10.4 (2.15)	10.1 (2.14)	10.2 (2.52)	0.200
PLT	195 (103)	211 (102)	193 (101)	182 (105)	0.001
RDW	15.7 (2.68)	15.1 (2.22)	15.8 (2.95)	16.1 (2.73)	<0.001
RBC	3.45 (0.81)	3.53 (0.77)	3.42 (0.77)	3.41 (0.88)	0.095
WBC	14.5 (12.0)	12.1 (7.15)	13.8 (7.32)	17.6 (17.6)	<0.001
Neu	11.5 (7.61)	9.43 (5.29)	11.2 (6.85)	13.8 (9.45)	<0.001
ALB	2.96 (0.57)	3.20 (0.49)	2.92 (0.56)	2.75 (0.56)	<0.001
AG	15.6 (4.74)	14.5 (4.02)	15.2 (4.63)	17.1 (5.11)	<0.001
Ca	8.33 (0.89)	8.55 (0.71)	8.29 (0.85)	8.16 (1.05)	<0.001
Cl	103 (7.72)	103 (7.15)	103 (7.68)	104 (8.29)	0.593
GLU	163 (89.9)	144 (64.1)	163 (79.5)	181 (115)	<0.001
K	4.25 (0.78)	4.22 (0.66)	4.25 (0.75)	4.27 (0.90)	0.710
Na	138 (6.63)	138 (6.31)	138 (6.67)	139 (6.90)	0.973
TCO_2_	23.7 (5.75)	24.9 (5.50)	24.5 (5.72)	21.5 (5.42)	<0.001
FCa	1.12 (0.12)	1.15 (0.11)	1.11 (0.11)	1.10 (0.14)	<0.001
Lac	2.33 (1.58)	1.13 (0.28)	1.89 (0.44)	3.95 (1.73)	<0.001
PCO_2_	41.5 (11.1)	42.5 (11.7)	42.0 (10.9)	39.8 (10.6)	0.002
PH	7.35 (0.10)	7.37 (0.09)	7.36 (0.09)	7.33 (0.10)	<0.001
PO_2_	112 (94.7)	119 (96.3)	111 (92.7)	105 (94.9)	0.177
INR	1.60 (0.90)	1.46 (0.84)	1.54 (0.68)	1.79 (1.09)	<0.001
PT	17.5 (10.5)	15.8 (9.12)	16.8 (7.48)	19.8 (13.6)	<0.001
PTT	38.8 (24.3)	36.6 (22.5)	37.5 (21.0)	42.2 (28.4)	0.011
ALT	150 (576)	91.3 (396)	136 (606)	223 (678)	0.007
AST	275 (1254)	168 (850)	256 (1401)	399 (1411)	0.031
TB	2.28 (5.00)	1.25 (3.14)	2.60 (5.78)	2.97 (5.45)	<0.001
CRE	1.76 (1.64)	1.70 (1.70)	1.77 (1.71)	1.79 (1.50)	0.755
URE	35.6 (28.6)	33.5 (28.4)	36.1 (28.6)	37.3 (28.7)	0.192
LDH	572 (1406)	406 (701)	549 (1363)	758 (1871)	0.002
CRRT	123 (11.7%)	33 (9.54%)	43 (12.1%)	47 (13.3%)	0.285
Ventilation	952 (90.2%)	305 (88.2%)	323 (90.7%)	324 (91.8%)	0.251
SA	766 (72.6%)	231 (66.8%)	263 (73.9%)	272 (77.1%)	0.008
VP	766 (72.6%)	225 (65.0%)	259 (72.8%)	282 (79.9%)	<0.001
GC	350 (33.2%)	115 (33.2%)	109 (30.6%)	126 (35.7%)	0.357
Hosp. time	22.5 (22.8)	24.2 (23.3)	20.7 (22.3)	22.6 (22.8)	0.124
Hosp. dead	202 (19.1%)	34 (9.83%)	72 (20.2%)	96 (27.2%)	<0.001
ICU time	8.41 (8.89)	8.87 (8.66)	8.21 (8.88)	8.16 (9.14)	0.494
ICU dead	217 (20.6%)	36 (10.4%)	78 (21.9%)	103 (29.2%)	<0.001

HTN, hyperlipidemia; AKI, acute kidney injury; CKD, chronic kidney disease; DM, diabetes; HLD, hyperlipidemia; HF, heart failure; IHD, ischemic heart disease; COPD, chronic obstructive pulmonary disease; SOFA, Sequential Organ Failure Assessment; APSIII, Acute Physiology and Chronic Health III Score; Charlson, Charlson’s comorbidity index score; SAPSII, Simplified Acute Physiology Score II; OASIS, Oxford Acute Severity of Illness Score; APACHII, Acute Physiology and Chronic Health Evaluation II; HR, heart rate; NBPS, non-invasive blood pressure systolic; RR, respiratory rate; NBPD, non-invasive diastolic blood pressure; SPO_2_, oxygen saturation; HCT, hematocrit; Hb, hemoglobin; PLT, platelet; RDW, red blood cell distribution width; RBC, red blood cell count; WBC, white blood cell; ALB, albumin; AG, anion gap; Glu, glucose; K, blood potassium; Na, blood sodium; Mg, blood magnesium; TCO_2_, total amount of carbon dioxide; PCO_2_, partial pressure of carbon dioxide; Lac, lactate; PH, acidity and alkalinity; PO_2_, oxygen partial pressure; INR, international normalized ratio; PT, prothrombin time; APTT, activated partial thromboplastin time; ALT, alanine aminotransferase; AST, aspartate aminotransferase; TB, total bilirubin; CRE, creatinine; UREA, urea nitrogen; CRRT, continuous renal replacement therapy; Ventilation, mechanical ventilation; GC, corticosteroids; VP, vasoactive drugs; Sa, analgesic and sedative drugs; SA, sedatives and analgesics.

### Association between LAR and short-term mortality

As shown in Table 2, a higher LAR was significantly associated with increased 28-day ICU mortality across all three Cox regression models: the unadjusted Model 1 (HR = 1.59, 95% CI: 1.39–1.81, *p* < 0.001), Model 2 adjusted for demographics and baseline comorbidities (HR = 1.48, 95% CI: 1.29–1.70, *p* < 0.001), and Model 3 further adjusted for intergroup differential variables (HR = 1.35, 95% CI: 1.08–1.68, *p* = 0.008). Similarly, compared to the low LAR group, the high LAR group exhibited a significantly increased risk of mortality in all three models: Model 1 (HR = 3.18, 95% CI: 2.17–4.65, *p* < 0.001; *p* for trend <0.001), Model 2 (HR = 2.80, 95% CI: 1.91–4.12, *p* < 0.001; *p* for trend <0.001), and Model 3 (HR = 1.73, 95% CI: 1.10–2.73, *p* = 0.018; *p* for trend <0.001).

**Table 2:** The relationship between LAR and short-term mortality rate in ICU (discovery queue).

	Model 1			Model 2			Model 3		
Characteristic	HR	95% CI	*p*-value	HR	95% CI	*p*-value	HR	95% CI	*p*-value
LAR	1.59	1.39, 1.81	<0.001	1.48	1.29, 1.70	<0.001	1.35	1.08, 1.68	0.008
LAR group									
Low	Ref	Ref		Ref	Ref		Ref	Ref	
Moderate	2.34	1.58, 3.48	<0.001	2.13	1.43, 3.17	<0.001	1.64	1.07, 2.50	0.023
High	3.18	2.17, 4.65	<0.001	2.80	1.91, 4.12	<0.001	1.73	1.10, 2.73	0.018
*p* for trend		<0.001			<0.001			<0.001	

CI, confidence interval; HR, hazard ratio.

**Figure 1:**
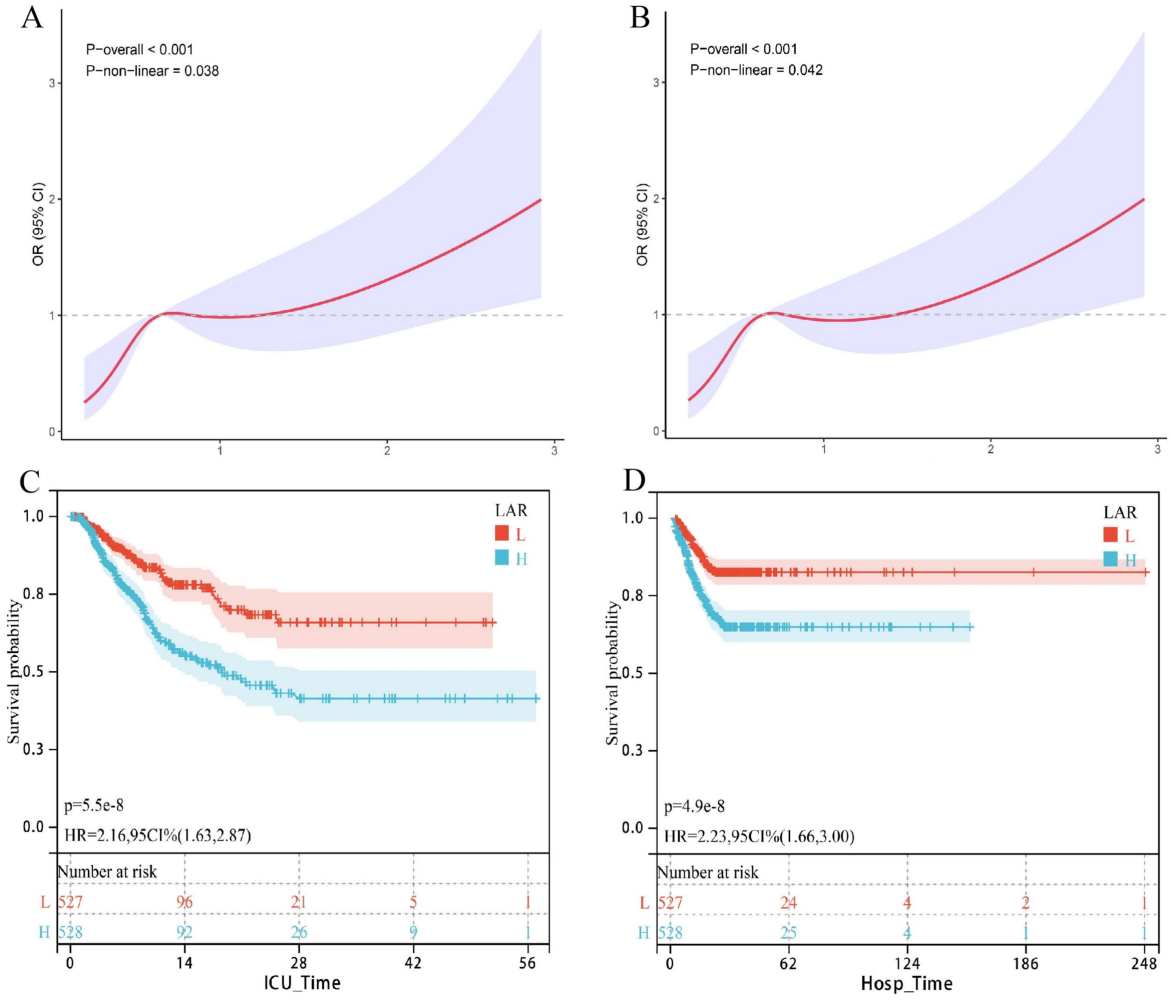
The correlation between LAR and short-term mortality in patients with urinary sepsis (internal discovery queue); association between LAR and short-term mortality in the internal discovery cohort. Restricted cubic spline curves show a significant nonlinear positive dose–response relationship between LAR and ICU **(A)** and in-hospital **(B)** mortality. Kaplan–Meier survival curves demonstrate significantly lower survival rates in patients with higher LAR for both ICU **(C)** and in-hospital **(D)** mortality.

Likewise, as presented in Table 3, a higher LAR consistently indicated an elevated risk of in-hospital mortality across all models: Model 1 (HR = 1.70, 95% CI: 1.47–1.97, *p* < 0.001), Model 2 (HR = 1.63, 95% CI: 1.40–1.89, *p* < 0.001), and Model 3 (HR = 1.50, 95% CI: 1.19–1.90, *p* = 0.001). The high LAR group also consistently demonstrated higher mortality compared to the low LAR group: Model 1 (HR = 2.98, 95% CI: 2.01–4.40, *p* < 0.001; *p* for trend <0.001), Model 2 (HR = 2.74, 95% CI: 1.84–4.07, *p* < 0.001; *p* for trend < 0.001), and Model 3 (HR = 1.71, 95% CI: 1.08–2.72, *p* = 0.022; *p* for trend <0.001). Restricted cubic spline (RCS) curves further revealed a non-linear positive dose–response relationship between LAR and short-term mortality in critically ill urosepsis patients (Figures 1A,B). Kaplan–Meier survival analysis similarly showed that higher LAR was associated with lower short-term survival rates (Figures 1C,D).

**Table 3:** The relationship between LAR and short-term mortality rate in hospital (discovery queue).

	Model 1			Model 2			Model 3		
Characteristic	HR	95% CI	*p*-value	HR	95% CI	*p*-value	HR	95% CI	*p*-value
LAR	1.70	1.47, 1.97	<0.001	1.63	1.40, 1.89	<0.001	1.50	1.19, 1.90	<0.001
LAR group									
Low	Ref	Ref		Ref	Ref		Ref	Ref	
Moderate	2.25	1.49, 3.38	<0.001	2.07	1.37, 3.11	<0.001	1.62	1.06, 2.48	0.027
High	2.98	2.01, 4.40	<0.001	2.74	1.84, 4.07	<0.001	1.71	1.08, 2.72	0.022
*p* for trend		<0.001			<0.001			<0.001	

CI, confidence interval; HR, hazard ratio.

### Incremental effect of LAR

**Figure 2:**
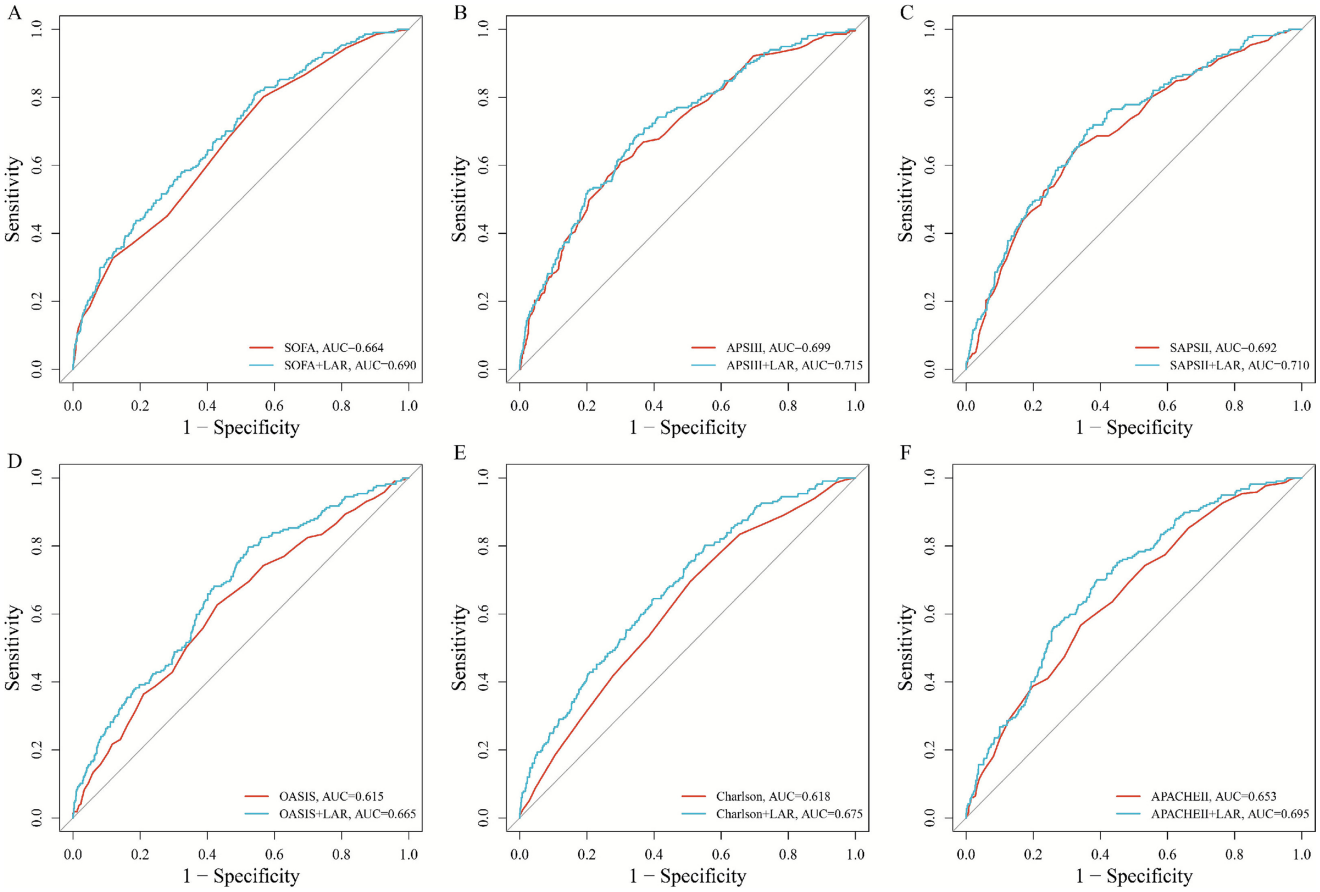
The incremental effect of LAR; adding LAR to six established severity scores (SOFA, APS III, SAPS II, OASIS, Charlson, APACHE II) consistently improved their discriminatory performance, as shown by increased area under the receiver operating characteristic curves **(A–F)**.

To evaluate the added value of the LAR, we tested its combination with six established severity scores (SOFA, APS III, SAPS II, OASIS, Charlson, APACHE II) for predicting 28-day ICU mortality. The inclusion of LAR universally enhanced the predictive accuracy of all models (Figures 2A–F), with DeLong’s test confirming statistically significant improvements for five of the six scores (SOFA: AUC 0.664 to 0.690, *p* = 0.004; SAPS II: 0.692 to 0.710, *p* = 0.017; OASIS: 0.615 to 0.665, *p* = 0.001; Charlson: 0.618 to 0.675, *p* < 0.001; APACHE II: 0.653 to 0.695, p < 0.001). A positive, albeit non-significant, increase was also observed for APS III (0.699 to 0.712, *p* = 0.058).

### Subgroup and interaction analysis

We conducted subgroup analyses and interaction tests to investigate whether demographic characteristics and comorbidities influenced the association between LAR and 28-day ICU/in-hospital mortality. After multivariable adjustment, the results (Supplementary Figures S1, S2) indicated that the association between LAR and 28-day ICU/in-hospital mortality did not reach statistical significance in certain subgroups. These included patients with comorbidities (e.g., hypertension, chronic kidney disease, diabetes, hyperlipidemia, heart failure, and chronic obstructive pulmonary disease) as well as the subgroup of older male patients. It is important to note that these specific subgroups had relatively smaller sample sizes and lower event rates, which may have resulted in insufficient statistical power. Therefore, the non-significant associations observed in these subgroup analyses should be interpreted with caution, as they may reflect a risk of type II error rather than a true absence of effect. The interaction tests identified a significant interaction effect only between age and LAR for ICU mortality (*p* for interaction <0.05), which further supports that the non-significant results in other subgroups are likely primarily limited by sample size constraints.

### External cohort validation

**Figure 3:**
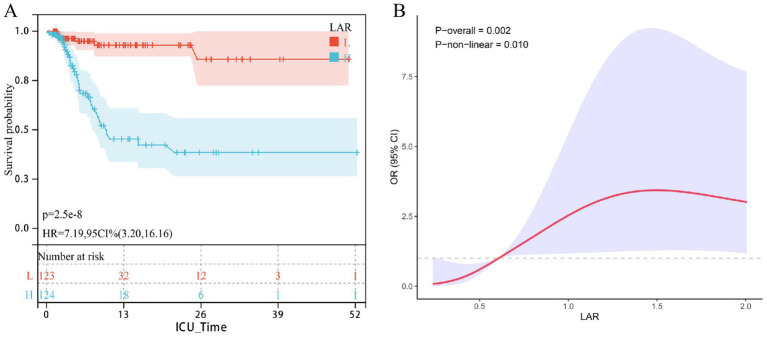
The correlation between LAR and short-term mortality in patients with urinary sepsis (external reality queue); Kaplan–Meier analysis **(A)** confirms significantly higher mortality in patients with LAR above the median. The restricted cubic spline curve **(B)** reveals a significant nonlinear positive dose–response relationship between LAR and ICU mortality.

The external validation cohort included 245 patients with urosepsis (28-day mortality = 14.28%). In the Cox regression model adjusted for all potential covariates, a higher LAR was significantly associated with an increased risk of 28-day mortality (HR = 2.12; 95% CI: 1.32–3.41, *p* = 0.002). Subsequent Kaplan–Meier analysis indicated that patients with LAR scores above the cohort median had significantly higher short-term mortality (log-rank *p* < 0.001; HR = 7.19, 95% CI: 3.20–16.16; Figure 3A). Furthermore, the RCS curve confirmed a significant non-linear positive dose–response relationship between LAR and ICU short-term mortality in the external cohort (Figure 3B).

### Development of an LAR-associated risk prediction model

**Figure 4:**
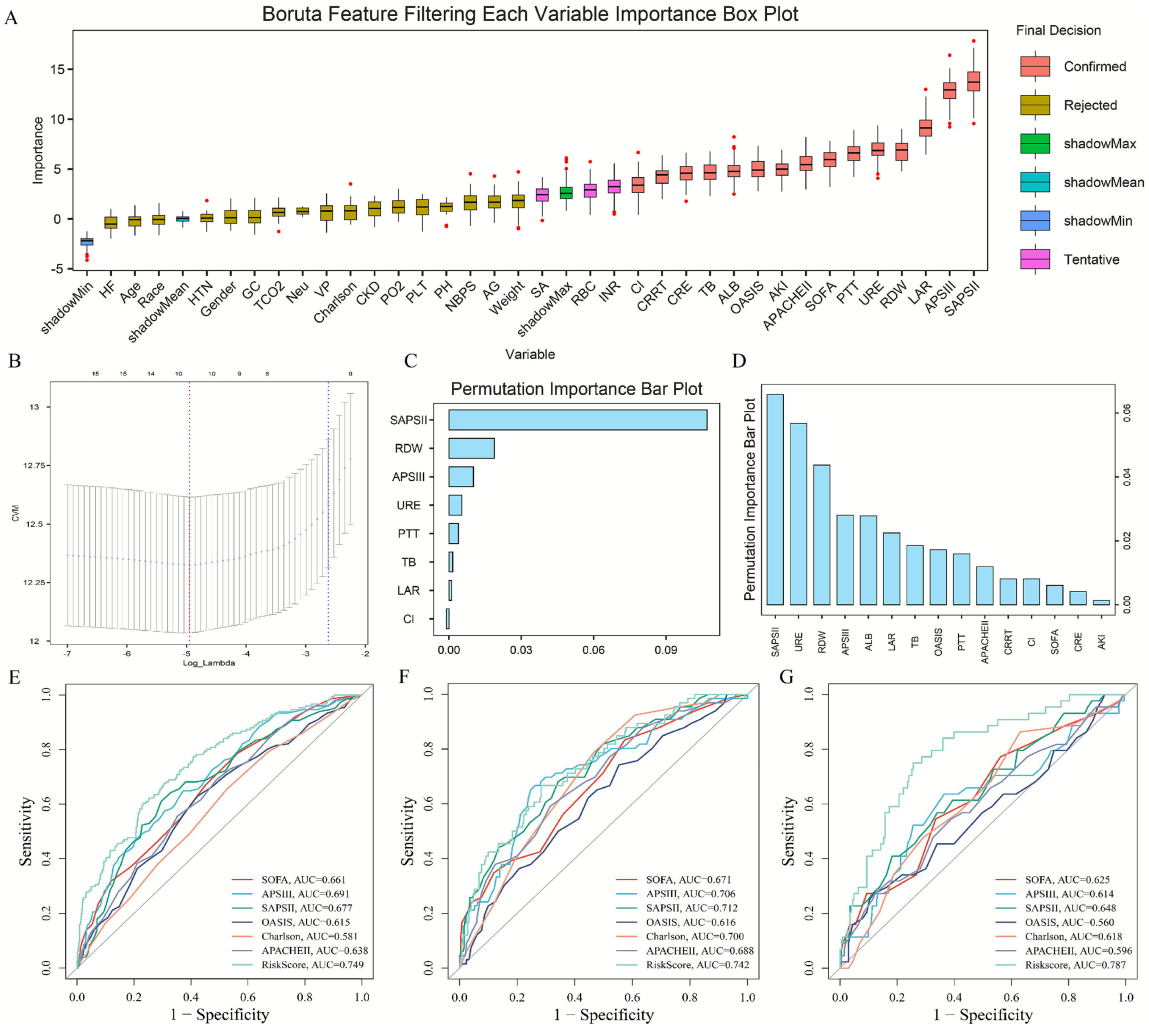
Establishment and evaluation of LAR related prediction analysis model; feature selection process using the Boruta algorithm **(A)**, random forest **(B)**, gradient boosting machine **(C)**, and LASSO regression **(D)** to identify the six final predictors. **(E–G)** The model comprising LAR, SAPS II, RDW, PTT, TB, and URE showed superior and stable predictive performance for 28-day ICU mortality across the internal training **(E)**, internal validation **(F)**, and external real-world **(G)** cohorts.

**Figure 5:**
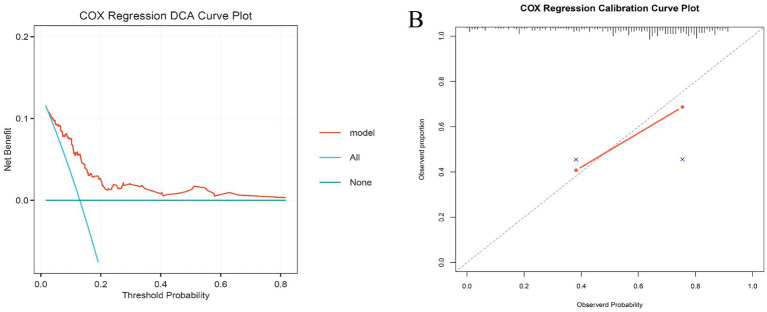
DCA curve and correction curve. **(A)** The calibration curve indicates good agreement between predicted and observed probabilities of 28-day ICU mortality. **(B)** Decision curve analysis demonstrates the positive net clinical benefit of using the model across a wide range of threshold probabilities.

In the internal training cohort, the Boruta algorithm identified 15 variables associated with ICU mortality (Figure 4A). Subsequently, random forest (Figure 4B), gradient boosting machine (feature importance score >0.01) (Figure 4C), and LASSO regression (lambda min = −4.947) (Figure 4D) collectively identified six key variables: LAR, SAPS II score, RDW, PTT, TB, and URE (blood urea nitrogen). These were used to construct a risk assessment model for predicting 28-day ICU mortality in critically ill urosepsis patients. The model formula is: Risk Score = (0.458617638 × LAR) + (0.015481692 × SAPSII) + (0.087598812 × RDW) + (0.004594067 × PTT) + (0.011365337 × TB) + (0.004909906 × URE). Compared to traditional severity scoring systems (SOFA, APS III, SAPS II, OASIS, Charlson, APACHE II), this model demonstrated superior sensitivity and specificity in predicting 28-day ICU mortality (Figures 4E–G). The AUC values were 0.749 in the internal training cohort (Figure 4E), 0.742 in the internal validation cohort (Figure 4F), and 0.787 in the external real-world cohort (Figure 4G). These consistent results indicate that the model possesses robust stability and predictive performance across diverse cohorts. The calibration curve and decision curve analysis further demonstrated the robustness and predictive accuracy of the analytical model (Figures 5A,B).

## Discussion

Urosepsis accounts for 9–31% of all sepsis cases, and its incidence continues to rise, posing a significant clinical and public health challenge (1–7). Therefore, identifying reliable and clinically applicable biomarkers to improve patient outcomes is crucial (25–27). In this context, an elevated LAR (lactate-to-albumin ratio) effectively integrates two cores, interconnected, and amplifying pathophysiological processes characteristic of the condition: “hypoxia” and “systemic inflammation.” On one hand, elevated lactate levels not only stem from systemic microcirculatory dysfunction in septic shock but also have specific relevance in the setting of urinary tract infections. Local inflammatory edema of the renal parenchyma due to pyelonephritis, as well as a sudden increase in intrarenal pressure and compromised blood flow caused by urinary tract obstruction (e.g., impacted stones), can directly lead to severe local tissue hypoxia and anaerobic metabolism within the kidney, thereby significantly elevating lactate levels (15, 28).

On the other hand, hypoalbuminemia is a sensitive marker for the severity of the systemic inflammatory response. Pathogen-associated molecular patterns (PAMPs) released during urinary tract infections can trigger a dysregulated inflammatory cascade, culminating in a “cytokine storm.” This state potently suppresses hepatic albumin synthesis and increases its extravascular leakage (8–10, 26, 29). Thus, an elevated LAR value effectively couples the tissue perfusion injury and metabolic crisis (hyperlactatemia) initiated by the local infectious focus with the resultant systemic inflammation (hypoalbuminemia). These two processes synergistically heighten the risk of organ dysfunction, providing a more specific pathophysiological basis for risk stratification.

Hypoalbuminemia, as a key component of LAR, holds significance far beyond a simple nutritional metric. It essentially represents and drives the core metabolic manifestation of a vicious cycle of “inflammatory-nutritional depletion.” Sustained systemic inflammation, mediated by pro-inflammatory cytokine signaling, directly suppresses the gene expression responsible for hepatic albumin synthesis, leading to hypoalbuminemia, which itself is a prominent feature of protein-energy malnutrition (8–10, 30, 31). Hypoalbuminemia, in turn, adversely affects the body through multiple pathways: the resultant decrease in colloid osmotic pressure exacerbates tissue edema, further impairing microcirculation; its diminished antioxidant capacity leaves tissues more vulnerable to oxidative stress damage; and more importantly, it signals that the body has entered a catabolism-dominant, depleted state where immune cells suffer from inadequate energy and material supply, leading to impaired function (32–35).

In the specific context of urosepsis, this cycle is particularly destructive. The systemic malnutrition and immune dysregulation marked by hypoalbuminemia significantly weaken the local mucosal immunity of the urinary tract. Specifically, it compromises the ability of urothelial cells to synthesize and maintain their protective surface glycoprotein layer (uromodulin), thinning the physical barrier. Concurrently, immunoglobulin A secretion may be reduced, and cellular immune responses may be delayed (36, 37). Furthermore, malnutrition-associated metabolic disturbances can alter urine composition (pH and organic acid concentration), creating a microenvironment more conducive to the adhesion, colonization, and expression of virulence factors (P-fimbriae) by pathogen such as *Escherichia coli* (38–40). This makes initial infection more likely to occur and harder for local defense mechanisms to clear, thereby substantially increasing the risk of ascending infection and the development of systemic sepsis.

In summary, an elevated LAR in patients with urosepsis serves as an efficient integrative biomarker for the synergistic and deleterious cascade of “local obstruction/infection-induced hypoxia to systemic inflammatory storm to secondary nutritional-metabolic exhaustion and barrier collapse.” It organically synthesizes information across three levels: the predisposing trigger (local perfusion/hypoxia), the core pathology (dysregulated inflammation), and the outcome amplifier (nutritional-immune exhaustion), thereby providing a more comprehensive pathophysiological snapshot than single biomarkers or traditional scoring systems.

This study is the first to demonstrate in the specific population of urosepsis that a high LAR is a strong and independent risk factor for short-term mortality. The clinical prediction model developed based on LAR shows potential advantages in identifying high-risk patients, serving as a supplement to traditional critical illness scores such as SOFA and APACHE II. These findings support the use of LAR as a practical early warning tool. Its significance lies in the early identification of high-risk urosepsis patients who may be trapped in the vicious cycle of “hypoxia-inflammation-exhaustion,” thereby prompting clinicians to enhance monitoring and initiate or adjust comprehensive management strategies, including infection source control, early resuscitation, and organ function support, more proactively. It is important to clarify that LAR itself does not indicate specific treatment regimens (such as the choice of particular antibiotics or vasoactive agents), but rather provides additional risk information regarding the overall severity of the patient’s physiological disturbances. Its core value lies in the fact that its components (lactate and albumin) are routinely measured in the ICU, enabling rapid acquisition at minimal additional cost, thus offering an easily integrated composite indicator for early risk stratification.

In subgroup analyses, the relationship between LAR and short-term mortality in urosepsis patients showed some heterogeneity across different demographic subgroups. Interaction analysis revealed a significant interaction effect only between age and LAR; no significant interactions were observed with other demographic or comorbidity factors. We speculate that the inconsistent strength of association in some subgroups might be related to reduced statistical power due to smaller sample sizes. Additionally, albumin levels are easily influenced by factors such as chronic diseases and aging, which might partly explain the variation in the LAR-mortality relationship across different patient groups.

While this study provides clinically meaningful findings and develops a potentially useful risk prediction model, several limitations of this study warrant careful consideration. First, the external validation cohort had a limited sample size and was derived from a single center, which constrains the generalizability of the findings. Inherent differences between the validation and derivation cohorts, including demographic characteristics (Asian vs. Western), regional clinical practices, and specific treatment protocols, may affect the model’s performance in different settings. Second, although multivariable models were used for adjustment, certain key, unmeasured treatment details (such as the precise timing of antimicrobial therapy and individualized fluid resuscitation strategies) could directly influence lactate and albumin levels, representing potential sources of residual confounding.

Third, due to the retrospective nature of this study, systematic measurements of infection-related biomarkers such as procalcitonin (PCT) and C-reactive protein (CRP) were unavailable. Therefore, we were unable to evaluate the incremental predictive value of LAR relative to these markers. Fourth, LAR was calculated based on a single static measurement at admission, precluding an assessment of its dynamic changes during treatment and their prognostic value. Finally, the retrospective observational design cannot establish causality, and the evolution of sepsis definitions along with potential incompleteness in confirming the infection source may introduce heterogeneity into the study population. Furthermore, although the addition of LAR to existing scoring systems showed a statistically significant improvement in AUC, the specific benefit of this incremental value in routine clinical practice still requires final confirmation through prospective, interventional studies. Therefore, to further clarify the potential clinical utility of LAR in patients with urosepsis, future large-scale, multicenter, prospective studies that integrate detailed treatment data and dynamic biomarker monitoring are needed to validate LAR and translate it into clinical practice.

## Conclusion

In summary, the LAR serves as a cost-effective, potential supplementary predictor of short-term mortality risk in critically ill patients with urosepsis. Its primary clinical value lies in the early identification of high-risk patients, thereby prompting closer monitoring and more proactive comprehensive therapeutic management. The pathway for its integration into clinical practice and its ultimate impact on patient outcomes await further clarification through prospective studies.

## Data Availability

The original contributions presented in the study are included in the article/Supplementary material, further inquiries can be directed to the corresponding authors.
