# Supplementary figures and images for: The lactate-to-albumin ratio as a potential biomarker for short-term mortality risk in critically ill patients with urosepsis: a retrospective study with dual-cohort validation
Source: Front Nutr. 2026 Feb 17;13:1753403. doi: 10.3389/fnut.2026.1753403 (PMC12953085; doi:10.3389/fnut.2026.1753403)

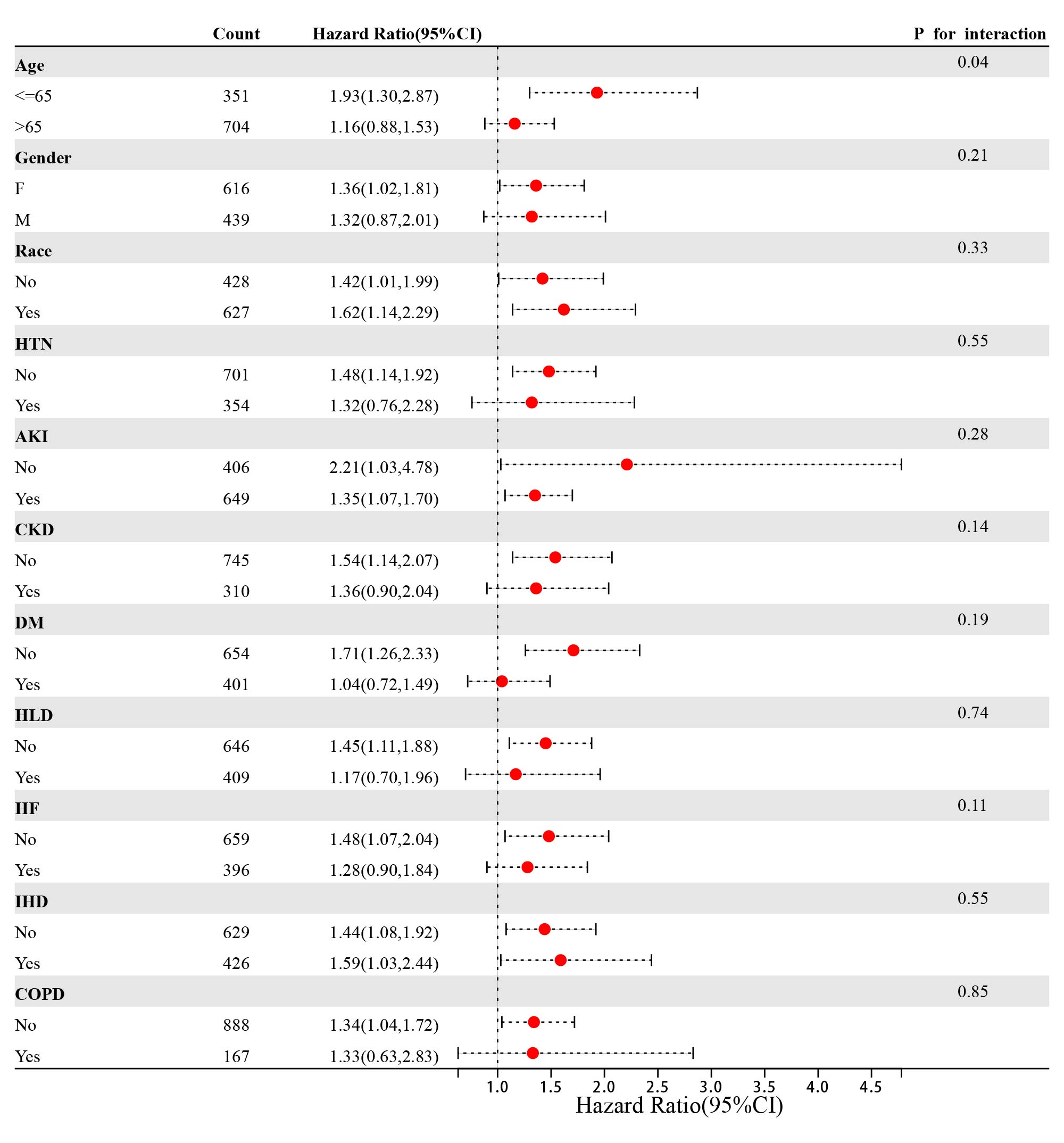

Supplement: Supplementary file 1 [file Image_1.jpg]

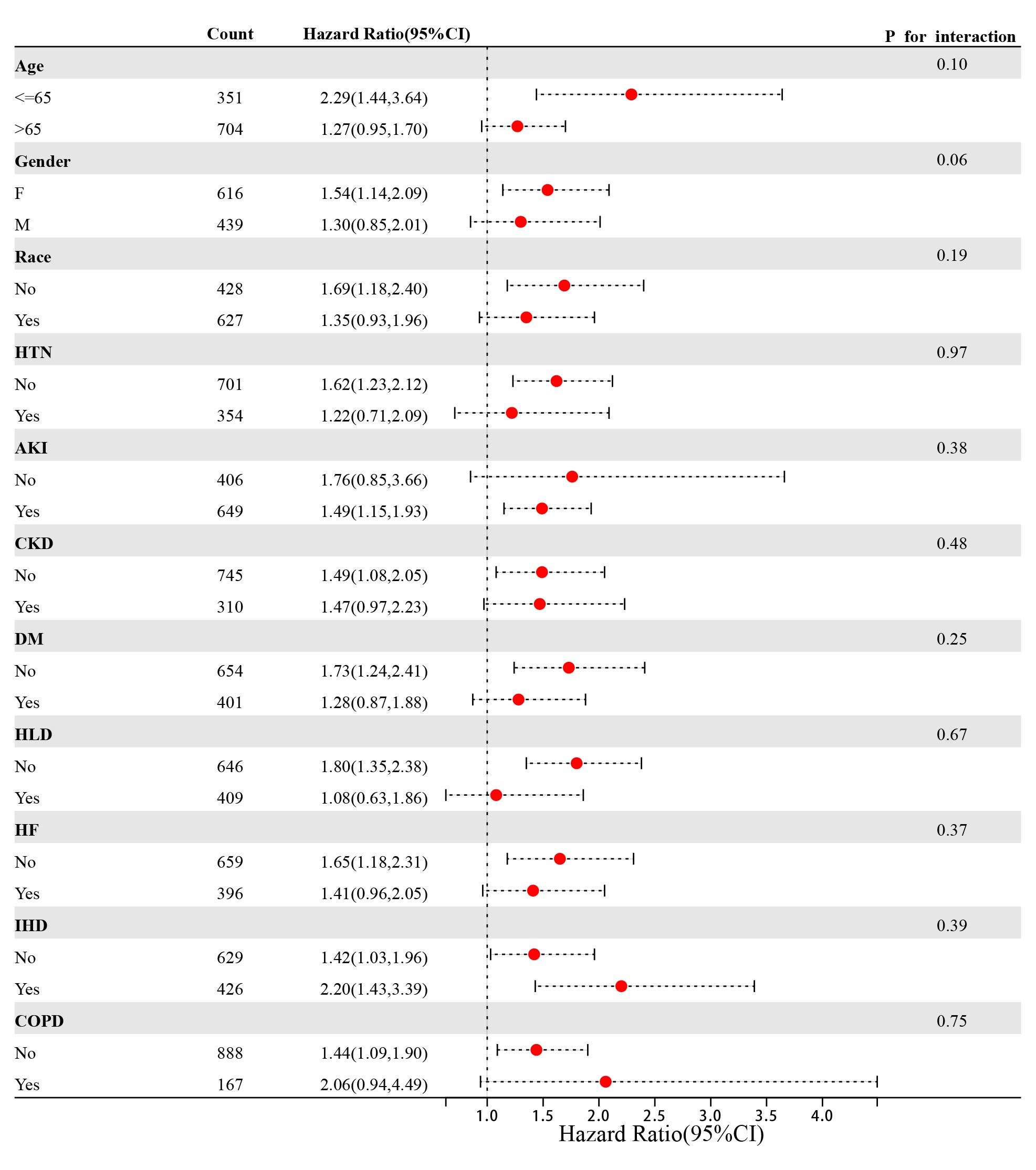

Supplement: Supplementary file 2 [file Image_2.jpg]
