# Supplementary material for: The lactate-to-albumin ratio as a potential biomarker for short-term mortality risk in critically ill patients with urosepsis: a retrospective study with dual-cohort validation
Source: Front Nutr. 2026 Feb 17;13:1753403. doi: 10.3389/fnut.2026.1753403 (PMC12953085; doi:10.3389/fnut.2026.1753403)
Supplement: Supplementary file 3 [file Table_1.docx]

Table S1: Baseline data of patients in ICU Dead (Discovery queue)

|  | **ALL** | **Survivor** | **No-Survivor** | **P-value** |
| --- | --- | --- | --- | --- |
|  | ***N=1055*** | ***N=838*** | ***N=217*** |  |
| Age | 70.5 (15.7) | 70.0 (15.8) | 72.4 (15.2) | **0.039** |
| Gender: | 439 (41.6%) | 350 (41.8%) | 89 (41.0%) | 0.902 |
| Race: | 627 (59.4%) | 499 (59.5%) | 128 (59.0%) | 0.942 |
| Weight | 82.2 (25.5) | 81.8 (24.7) | 83.7 (28.5) | 0.374 |
| HTN: | 354 (33.6%) | 304 (36.3%) | 50 (23.0%) | **<0.001** |
| AKI: | 649 (61.5%) | 472 (56.3%) | 177 (81.6%) | **<0.001** |
| CKD: | 310 (29.4%) | 233 (27.8%) | 77 (35.5%) | **0.033** |
| DM: | 401 (38.0%) | 311 (37.1%) | 90 (41.5%) | 0.271 |
| HLD: | 409 (38.8%) | 339 (40.5%) | 70 (32.3%) | **0.033** |
| HF: | 396 (37.5%) | 296 (35.3%) | 100 (46.1%) | **0.005** |
| IHD: | 426 (40.4%) | 334 (39.9%) | 92 (42.4%) | 0.547 |
| COPD: | 167 (15.8%) | 128 (15.3%) | 39 (18.0%) | 0.386 |
| SOFA | 7.06 (3.58) | 6.59 (3.27) | 8.91 (4.10) | **<0.001** |
| APSIII | 56.8 (21.8) | 53.5 (20.0) | 69.3 (24.0) | **<0.001** |
| SAPSII | 44.0 (13.1) | 42.2 (12.4) | 51.0 (13.2) | **<0.001** |
| OASIS | 35.9 (8.42) | 35.2 (8.26) | 38.6 (8.52) | **<0.001** |
| Charlson | 6.03 (2.86) | 5.78 (2.82) | 6.97 (2.81) | **<0.001** |
| APACHEII | 21.6 (7.12) | 20.8 (6.91) | 24.7 (7.10) | **<0.001** |
| HR | 90.6 (20.7) | 90.2 (20.6) | 92.1 (21.1) | 0.224 |
| NBPS | 119 (24.3) | 120 (24.5) | 116 (22.9) | **0.021** |
| NBPD | 69.3 (20.2) | 69.5 (20.0) | 68.5 (21.0) | 0.547 |
| NBPM | 82.4 (19.8) | 82.8 (19.8) | 81.1 (19.9) | 0.259 |
| RR | 20.5 (6.66) | 20.4 (6.68) | 21.0 (6.58) | 0.234 |
| Spo2 | 96.6 (4.56) | 96.6 (4.66) | 96.3 (4.14) | 0.264 |
| Lym | 1.55 (5.58) | 1.44 (4.29) | 1.97 (8.97) | 0.394 |
| HCT | 31.8 (6.94) | 32.0 (6.88) | 31.0 (7.15) | 0.069 |
| Hb | 10.2 (2.28) | 10.3 (2.27) | 9.95 (2.29) | **0.045** |
| PLT | 195 (103) | 199 (102) | 183 (108) | **0.049** |
| RDW | 15.7 (2.68) | 15.4 (2.40) | 16.9 (3.28) | **<0.001** |
| RBC | 3.45 (0.81) | 3.48 (0.79) | 3.34 (0.87) | **0.041** |
| WBC | 14.5 (12.0) | 14.0 (9.22) | 16.4 (19.1) | 0.077 |
| Neu | 11.5 (7.61) | 11.1 (7.34) | 12.9 (8.46) | **0.005** |
| ALB | 2.96 (0.57) | 2.99 (0.54) | 2.84 (0.64) | **0.001** |
| AG | 15.6 (4.74) | 15.3 (4.57) | 16.7 (5.19) | **<0.001** |
| Ca | 8.33 (0.89) | 8.34 (0.89) | 8.31 (0.92) | 0.646 |
| Cl | 103 (7.72) | 104 (7.38) | 102 (8.78) | **0.004** |
| GLU | 163 (89.9) | 162 (90.5) | 166 (87.5) | 0.484 |
| K | 4.25 (0.78) | 4.23 (0.77) | 4.33 (0.79) | 0.087 |
| Na | 138 (6.63) | 139 (6.29) | 138 (7.77) | 0.120 |
| TCO2 | 23.7 (5.75) | 23.8 (5.68) | 22.9 (5.96) | **0.037** |
| FCa | 1.12 (0.12) | 1.12 (0.12) | 1.10 (0.12) | 0.055 |
| Lac | 2.33 (1.58) | 2.17 (1.38) | 2.96 (2.09) | **<0.001** |
| PCO2 | 41.5 (11.1) | 41.4 (11.2) | 41.7 (10.9) | 0.697 |
| PH | 7.35 (0.10) | 7.36 (0.09) | 7.34 (0.10) | **0.001** |
| PO2 | 112 (94.7) | 117 (99.1) | 91.6 (72.2) | **<0.001** |
| INR | 1.60 (0.90) | 1.52 (0.76) | 1.91 (1.24) | **<0.001** |
| PT | 17.5 (10.5) | 16.6 (9.38) | 20.8 (13.6) | **<0.001** |
| PTT | 38.8 (24.3) | 37.4 (23.5) | 44.2 (26.5) | **0.001** |
| ALT | 150 (576) | 160 (618) | 113 (368) | 0.151 |
| AST | 275 (1254) | 295 (1367) | 198 (654) | 0.135 |
| TB | 2.28 (5.00) | 1.86 (4.14) | 3.90 (7.23) | **<0.001** |
| CRE | 1.76 (1.64) | 1.68 (1.59) | 2.05 (1.78) | **0.007** |
| URE | 35.6 (28.6) | 32.8 (26.1) | 46.5 (34.8) | **<0.001** |
| LDH | 572 (1406) | 560 (1372) | 619 (1533) | 0.609 |
| CRRT: | 123 (11.7%) | 74 (8.83%) | 49 (22.6%) | **<0.001** |
| Ventilation: | 952 (90.2%) | 753 (89.9%) | 199 (91.7%) | 0.491 |
| SA: | 766 (72.6%) | 591 (70.5%) | 175 (80.6%) | **0.004** |
| VP: | 766 (72.6%) | 582 (69.5%) | 184 (84.8%) | **<0.001** |
| GC: | 350 (33.2%) | 257 (30.7%) | 93 (42.9%) | **0.001** |
| LAR | 0.83 (0.65) | 0.76 (0.53) | 1.12 (0.93) | **<0.001** |
| LAR group: |  |  |  | **<0.001** |
| Low | 346 (32.8%) | 310 (37.0%) | 36 (16.6%) |  |
| Moderate | 356 (33.7%) | 278 (33.2%) | 78 (35.9%) |  |
| High | 353 (33.5%) | 250 (29.8%) | 103 (47.5%) |  |

Notes: HTN: Hyperlipidemia; AKI: Acute Kidney Injury; CKD: Chronic Kidney Disease; DM: Diabetes; HLD: hyperlipidemia; HF: Heart Failure; IHD: Ischemic Heart Disease; COPD：Chronic Obstructive Pulmonary Disease; SOFA：Sequential Organ Failure Assessment; APSIII: Acute Physiology and Chronic Health III Score; Charlson: Charlson's comorbidity index score; SAPSII：Simplified Acute Physiology Score II; OASIS：Oxford Acute Severity of Illness Score; APACHII：Acute Physiology and Chronic Health Evaluation II; HR：Heart Rate; NBPS：Non-invasive Blood Pressure Systolic; RR：Respiratory Rate; NBPD: Non-invasive diastolic blood pressure; SPO2: Oxygen saturation; HCT：Hematocrit; Hb：Hemoglobin; PLT：Platelet; RDW：Red Blood Cell Distribution Width; RBC: Red blood cell count; WBC：White Blood Cell; ALB：Albumin; AG：Anion Gap; Glu: Glucose; K：Blood potassium; Na：Blood sodium; Mg: Blood magnesium; TCO2: Total amount of carbon dioxide; PCO2: Partial pressure of carbon dioxide; Lac：Lactate; PH: acidity and alkalinity; PO2: Oxygen partial pressure; INR：International Normalized Ratio; PT：Prothrombin Time; APTT：Activated Partial Thromboplastin Time; ALT：Alanine Aminotransferase; AST：Aspartate Aminotransferase; TB：Total Bilirubin; CRE：Creatinine; UREA：Urea Nitrogen; CRRT：Continuous Renal Replacement Therapy; Ventilation：Mechanical Ventilation; GC: Corticosteroids; VP: Vasoactive drugs; Sa: Analgesic and sedative drugs; SA: Sedatives and analgesics.
