# Supplementary material for: The lactate-to-albumin ratio as a potential biomarker for short-term mortality risk in critically ill patients with urosepsis: a retrospective study with dual-cohort validation
Source: Front Nutr. 2026 Feb 17;13:1753403. doi: 10.3389/fnut.2026.1753403 (PMC12953085; doi:10.3389/fnut.2026.1753403)
Supplement: Supplementary file 4 [file Table_2.docx]

Table S2: Baseline data of patients in Hosp Dead (Discovery queue)

|  | **ALL** | **Survivor** | **No-survivor** | **P-value** |
| --- | --- | --- | --- | --- |
|  | ***N=1055*** | ***N=853*** | ***N=202*** |  |
| Age | 70.5 (15.7) | 69.9 (15.7) | 73.0 (15.3) | **0.012** |
| Gender: | 439 (41.6%) | 355 (41.6%) | 84 (41.6%) | 1.000 |
| Race: | 627 (59.4%) | 509 (59.7%) | 118 (58.4%) | 0.805 |
| Weight | 82.2 (25.5) | 81.9 (24.8) | 83.7 (28.5) | 0.413 |
| HTN: | 354 (33.6%) | 308 (36.1%) | 46 (22.8%) | **<0.001** |
| AKI: | 649 (61.5%) | 487 (57.1%) | 162 (80.2%) | **<0.001** |
| CKD: | 310 (29.4%) | 238 (27.9%) | 72 (35.6%) | **0.037** |
| DM: | 401 (38.0%) | 320 (37.5%) | 81 (40.1%) | 0.549 |
| HLD: | 409 (38.8%) | 342 (40.1%) | 67 (33.2%) | 0.083 |
| HF: | 396 (37.5%) | 304 (35.6%) | 92 (45.5%) | **0.011** |
| IHD: | 426 (40.4%) | 340 (39.9%) | 86 (42.6%) | 0.530 |
| COPD: | 167 (15.8%) | 130 (15.2%) | 37 (18.3%) | 0.332 |
| SOFA | 7.06 (3.58) | 6.65 (3.33) | 8.83 (4.02) | **<0.001** |
| APSIII | 56.8 (21.8) | 53.9 (20.3) | 68.8 (23.8) | **<0.001** |
| SAPSII | 44.0 (13.1) | 42.4 (12.6) | 50.7 (13.1) | **<0.001** |
| OASIS | 35.9 (8.42) | 35.2 (8.31) | 38.6 (8.39) | **<0.001** |
| Charlson | 6.03 (2.86) | 5.81 (2.84) | 6.93 (2.78) | **<0.001** |
| APACHEII | 21.6 (7.12) | 20.9 (6.99) | 24.5 (6.99) | **<0.001** |
| HR | 90.6 (20.7) | 90.3 (20.7) | 91.7 (20.7) | 0.413 |
| NBPS | 119 (24.3) | 120 (24.6) | 116 (22.3) | **0.021** |
| NBPD | 69.3 (20.2) | 69.5 (20.2) | 68.2 (20.2) | 0.415 |
| NBPM | 82.4 (19.8) | 82.8 (20.0) | 80.9 (19.2) | 0.208 |
| RR | 20.5 (6.66) | 20.4 (6.67) | 21.0 (6.58) | 0.258 |
| Spo2 | 96.6 (4.56) | 96.6 (4.65) | 96.4 (4.16) | 0.439 |
| Lym | 1.55 (5.58) | 1.43 (4.25) | 2.02 (9.29) | 0.382 |
| HCT | 31.8 (6.94) | 31.9 (6.91) | 31.3 (7.08) | 0.285 |
| Hb | 10.2 (2.28) | 10.3 (2.28) | 10.1 (2.27) | 0.210 |
| PLT | 195 (103) | 198 (102) | 185 (109) | 0.130 |
| RDW | 15.7 (2.68) | 15.4 (2.55) | 16.6 (2.98) | **<0.001** |
| RBC | 3.45 (0.81) | 3.47 (0.79) | 3.38 (0.86) | 0.173 |
| WBC | 14.5 (12.0) | 14.0 (9.21) | 16.5 (19.7) | 0.080 |
| Neu | 11.5 (7.61) | 11.1 (7.30) | 13.0 (8.68) | **0.005** |
| ALB | 2.96 (0.57) | 2.99 (0.55) | 2.84 (0.63) | **0.003** |
| AG | 15.6 (4.74) | 15.4 (4.70) | 16.6 (4.79) | **0.001** |
| Ca | 8.33 (0.89) | 8.35 (0.91) | 8.28 (0.80) | 0.319 |
| Cl | 103 (7.72) | 104 (7.50) | 102 (8.50) | **0.020** |
| GLU | 163 (89.9) | 162 (90.9) | 166 (85.6) | 0.503 |
| K | 4.25 (0.78) | 4.23 (0.77) | 4.35 (0.79) | 0.052 |
| Na | 138 (6.63) | 139 (6.30) | 138 (7.84) | 0.216 |
| TCO2 | 23.7 (5.75) | 23.8 (5.67) | 22.9 (6.06) | **0.039** |
| Fca | 1.12 (0.12) | 1.12 (0.12) | 1.10 (0.12) | **0.031** |
| Lac | 2.33 (1.58) | 2.18 (1.38) | 2.98 (2.13) | **<0.001** |
| PCO2 | 41.5 (11.1) | 41.5 (11.2) | 41.5 (10.7) | 0.999 |
| PH | 7.35 (0.10) | 7.36 (0.09) | 7.34 (0.10) | **0.003** |
| PO2 | 112 (94.7) | 116 (99.0) | 91.3 (70.6) | **<0.001** |
| INR | 1.60 (0.90) | 1.52 (0.77) | 1.91 (1.26) | **<0.001** |
| PT | 17.5 (10.5) | 16.7 (9.41) | 20.8 (13.9) | **<0.001** |
| PTT | 38.8 (24.3) | 37.6 (23.8) | 43.8 (25.4) | **0.002** |
| ALT | 150 (576) | 158 (613) | 118 (380) | 0.236 |
| AST | 275 (1254) | 292 (1355) | 206 (677) | 0.197 |
| TB | 2.28 (5.00) | 1.93 (4.31) | 3.78 (7.03) | **<0.001** |
| CRE | 1.76 (1.64) | 1.70 (1.61) | 2.02 (1.74) | **0.016** |
| URE | 35.6 (28.6) | 33.1 (26.4) | 46.3 (34.5) | **<0.001** |
| LDH | 572 (1406) | 559 (1360) | 629 (1586) | 0.560 |
| CRRT: | 123 (11.7%) | 79 (9.26%) | 44 (21.8%) | **<0.001** |
| Ventilation: | 952 (90.2%) | 766 (89.8%) | 186 (92.1%) | 0.396 |
| SA: | 766 (72.6%) | 605 (70.9%) | 161 (79.7%) | 0.015 |
| VP: | 766 (72.6%) | 597 (70.0%) | 169 (83.7%) | **<0.001** |
| GC: | 350 (33.2%) | 264 (30.9%) | 86 (42.6%) | **0.002** |
| LAR | 0.83 (0.65) | 0.76 (0.53) | 1.12 (0.95) | **<0.001** |
| LAR group: |  |  |  | **<0.001** |
| Low | 346 (32.8%) | 312 (36.6%) | 34 (16.8%) |  |
| Moderate | 356 (33.7%) | 284 (33.3%) | 72 (35.6%) |  |
| High | 353 (33.5%) | 257 (30.1%) | 96 (47.5%) |  |

Notes: HTN: Hyperlipidemia; AKI: Acute Kidney Injury; CKD: Chronic Kidney Disease; DM: Diabetes; HLD: hyperlipidemia; HF: Heart Failure; IHD: Ischemic Heart Disease; COPD：Chronic Obstructive Pulmonary Disease; SOFA：Sequential Organ Failure Assessment; APSIII: Acute Physiology and Chronic Health III Score; Charlson: Charlson's comorbidity index score; SAPSII：Simplified Acute Physiology Score II; OASIS：Oxford Acute Severity of Illness Score; APACHII：Acute Physiology and Chronic Health Evaluation II; HR：Heart Rate; NBPS：Non-invasive Blood Pressure Systolic; RR：Respiratory Rate; NBPD: Non-invasive diastolic blood pressure; SPO2: Oxygen saturation; HCT：Hematocrit; Hb：Hemoglobin; PLT：Platelet; RDW：Red Blood Cell Distribution Width; RBC: Red blood cell count; WBC：White Blood Cell; ALB：Albumin; AG：Anion Gap; Glu: Glucose; K：Blood potassium; Na：Blood sodium; Mg: Blood magnesium; TCO2: Total amount of carbon dioxide; PCO2: Partial pressure of carbon dioxide; Lac：Lactate; PH: acidity and alkalinity; PO2: Oxygen partial pressure; INR：International Normalized Ratio; PT：Prothrombin Time; APTT：Activated Partial Thromboplastin Time; ALT：Alanine Aminotransferase; AST：Aspartate Aminotransferase; TB：Total Bilirubin; CRE：Creatinine; UREA：Urea Nitrogen; CRRT：Continuous Renal Replacement Therapy; Ventilation：Mechanical Ventilation; GC: Corticosteroids; VP: Vasoactive drugs; Sa: Analgesic and sedative drugs; SA: Sedatives and analgesics.
