# Supplementary material for: The lactate-to-albumin ratio as a potential biomarker for short-term mortality risk in critically ill patients with urosepsis: a retrospective study with dual-cohort validation
Source: Front Nutr. 2026 Feb 17;13:1753403. doi: 10.3389/fnut.2026.1753403 (PMC12953085; doi:10.3389/fnut.2026.1753403)
Supplement: Supplementary file 5 [file Table_3.docx]

Table S3: Baseline data of patients in ICU Dead (Verify queue

|  | **ALL** | **Survivor** | **No-survivor** | **P-value** |
| --- | --- | --- | --- | --- |
|  | ***N=247*** | ***N=203*** | ***N=44*** |  |
| Age | 70.5 (15.6) | 69.3 (15.8) | 76.4 (13.7) | **0.003** |
| Gender: | 102 (41.3%) | 85 (41.9%) | 17 (38.6%) | 0.821 |
| BMI | 27.7 (4.63) | 27.6 (4.53) | 27.9 (5.14) | 0.761 |
| HTN: | 79 (32.0%) | 72 (35.5%) | 7 (15.9%) | **0.019** |
| AKI: | 137 (55.5%) | 101 (49.8%) | 36 (81.8%) | **<0.001** |
| CKD: | 68 (27.5%) | 50 (24.6%) | 18 (40.9%) | **0.045** |
| DM: | 93 (37.7%) | 73 (36.0%) | 20 (45.5%) | 0.314 |
| COPD: | 39 (15.8%) | 32 (15.8%) | 7 (15.9%) | 1.000 |
| SOFA | 6.63 (3.35) | 6.32 (3.10) | 8.09 (4.04) | **0.008** |
| APSIII | 54.8 (19.8) | 53.2 (18.1) | 62.2 (25.1) | **0.027** |
| SAPSII | 43.9 (12.8) | 42.5 (11.9) | 50.2 (14.6) | **0.002** |
| OASIS | 35.6 (8.34) | 35.3 (8.17) | 37.4 (8.97) | 0.158 |
| Charlson | 6.14 (2.99) | 5.95 (3.00) | 7.00 (2.80) | **0.029** |
| APACHEII | 20.7 (7.19) | 20.2 (6.82) | 23.0 (8.40) | **0.043** |
| HR | 90.5 (20.2) | 90.4 (20.2) | 90.6 (20.5) | 0.964 |
| NBPS | 119 (25.2) | 120 (25.6) | 116 (23.3) | 0.359 |
| NBPD | 69.1 (19.3) | 69.9 (18.7) | 65.3 (21.5) | 0.200 |
| NBPM | 82.4 (19.3) | 83.2 (19.0) | 78.6 (20.1) | 0.175 |
| RR | 20.0 (6.57) | 20.3 (6.50) | 18.5 (6.76) | 0.107 |
| Spo2 | 96.7 (4.39) | 96.7 (4.44) | 96.6 (4.24) | 0.901 |
| Lym | 1.19 (0.91) | 1.22 (0.88) | 1.09 (1.01) | 0.427 |
| HCT | 32.2 (6.99) | 32.4 (6.84) | 31.7 (7.69) | 0.579 |
| Hb | 10.4 (2.30) | 10.4 (2.26) | 10.2 (2.49) | 0.685 |
| PLT | 199 (101) | 209 (97.9) | 156 (102) | **0.003** |
| RDW | 15.7 (2.60) | 15.4 (2.32) | 17.1 (3.34) | **0.003** |
| RBC | 3.52 (0.80) | 3.55 (0.78) | 3.39 (0.88) | 0.297 |
| WBC | 13.8 (9.12) | 13.6 (9.49) | 14.6 (7.19) | 0.469 |
| Neu | 11.0 (6.93) | 10.6 (6.83) | 13.0 (7.18) | 0.054 |
| ALB | 2.95 (0.58) | 2.99 (0.56) | 2.78 (0.67) | 0.051 |
| AG | 15.1 (4.67) | 15.0 (4.54) | 15.9 (5.22) | 0.269 |
| Ca | 8.36 (0.91) | 8.37 (0.94) | 8.32 (0.78) | 0.731 |
| Cl | 103 (7.05) | 103 (6.87) | 100 (7.43) | **0.018** |
| GLU | 158 (80.8) | 160 (85.3) | 149 (55.5) | 0.308 |
| K | 4.20 (0.72) | 4.18 (0.72) | 4.29 (0.72) | 0.371 |
| Na | 138 (6.03) | 139 (5.57) | 137 (7.69) | 0.117 |
| Lac | 2.23 (1.57) | 2.04 (1.42) | 3.10 (1.93) | **0.001** |
| PCO2 | 42.6 (11.7) | 42.5 (11.9) | 43.0 (10.8) | 0.824 |
| PH | 7.35 (0.10) | 7.36 (0.10) | 7.34 (0.10) | 0.160 |
| PO2 | 109 (92.6) | 115 (97.4) | 78.2 (58.5) | **0.001** |
| INR | 1.54 (0.73) | 1.46 (0.63) | 1.90 (1.01) | **0.007** |
| PT | 17.1 (11.2) | 16.3 (11.1) | 20.7 (11.0) | **0.018** |
| PTT | 37.7 (22.2) | 36.5 (21.0) | 42.9 (26.6) | 0.143 |
| ALT | 143 (566) | 139 (565) | 161 (577) | 0.822 |
| AST | 281 (1407) | 282 (1469) | 271 (1092) | 0.955 |
| TB | 2.33 (5.38) | 1.96 (4.87) | 4.03 (7.10) | 0.071 |
| CRE | 1.62 (1.36) | 1.54 (1.38) | 1.98 (1.20) | **0.040** |
| URE | 36.4 (25.7) | 34.4 (25.9) | 45.8 (23.0) | **0.005** |
| LDH | 484 (1031) | 471 (1026) | 544 (1063) | 0.679 |
| CRRT: | 20 (8.10%) | 13 (6.40%) | 7 (15.9%) | 0.060 |
| Ventilation: | 214 (86.6%) | 175 (86.2%) | 39 (88.6%) | 0.853 |
| SA: | 182 (73.7%) | 148 (72.9%) | 34 (77.3%) | 0.684 |
| VP: | 185 (74.9%) | 147 (72.4%) | 38 (86.4%) | 0.081 |
| LAR | 0.78 (0.56) | 0.71 (0.52) | 1.13 (0.63) | **<0.001** |

Notes: HTN: Hyperlipidemia; AKI: Acute Kidney Injury; CKD: Chronic Kidney Disease; DM: Diabetes; HLD: hyperlipidemia; HF: Heart Failure; IHD: Ischemic Heart Disease; COPD：Chronic Obstructive Pulmonary Disease; BMI: Body mass index; SOFA：Sequential Organ Failure Assessment; APSIII: Acute Physiology and Chronic Health III Score; Charlson: Charlson's comorbidity index score; SAPSII：Simplified Acute Physiology Score II; OASIS：Oxford Acute Severity of Illness Score; APACHII：Acute Physiology and Chronic Health Evaluation II; HR：Heart Rate; NBPS：Non-invasive Blood Pressure Systolic; RR：Respiratory Rate; NBPD: Non-invasive diastolic blood pressure; SPO2: Oxygen saturation; HCT：Hematocrit; Hb：Hemoglobin; PLT：Platelet; RDW：Red Blood Cell Distribution Width; RBC: Red blood cell count; WBC：White Blood Cell; ALB：Albumin; AG：Anion Gap; Glu: Glucose; K：Blood potassium; Na：Blood sodium; Mg: Blood magnesium; TCO2: Total amount of carbon dioxide; PCO2: Partial pressure of carbon dioxide; Lac：Lactate; PH: acidity and alkalinity; PO2: Oxygen partial pressure; INR：International Normalized Ratio; PT：Prothrombin Time; APTT：Activated Partial Thromboplastin Time; ALT：Alanine Aminotransferase; AST：Aspartate Aminotransferase; TB：Total Bilirubin; CRE：Creatinine; UREA：Urea Nitrogen; CRRT：Continuous Renal Replacement Therapy; Ventilation：Mechanical Ventilation; VP: Vasoactive drugs; Sa: Analgesic and sedative drugs; SA: Sedatives and analgesics.
